# *In Vitro* and *In Vivo* Effects of Natural Putative Secretagogues of Glucagon-Like Peptide-1 (GLP-1)

**DOI:** 10.3797/scipharm.1104-16

**Published:** 2011-06-09

**Authors:** Eamon P. Rafferty, Alastair R. Wylie, Chris T. Elliott, Olivier P. Chevallier, David J. Grieve, Brian D. Green

**Affiliations:** 1 School of Biological Sciences, Queen’s University Belfast, BT9 5AG, Northern Ireland, United Kingdom; 2 Agri-Food and Biosciences Institute, Newforge Lane, BT9 5PX, Belfast, Northern Ireland, United Kingdom; 3 School of Medicine, Dentistry and Biomedical Sciences, Queen’s University Belfast, Royal Victoria Hospital, Grosvenor Road, Belfast, BT12 6BA, Northern Ireland, United Kingdom

**Keywords:** Diabetes, Glucose, Hormone, Secretion, Olive leaf extract, Glutamine, Casein, Chlorogenic acid

## Abstract

Glucagon-like peptide-1 (GLP-1) is an intestinal hormone with well-established glucose-lowering activity. The *in vitro* and *in vivo* actions of natural putative secretagogues of GLP-1 were investigated. The acute GLP-1 releasing activity of olive leaf extract (OLE), glutamine (GLN), alpha casein (ACAS), beta casein (BCAS) and chlorogenic acid (CGA) were assessed in STC-1 cells and C57BL/6 mice. All compounds except ACAS significantly increased acute *in vitro* GLP-1 secretion (66–386%; P<0.05–0.001). Oral gavage of OLE and GLN modestly increased plasma GLP-1 concentrations (48% and 41%, respectively), but did not lower glycaemic excursions. OLE and GLN are potent stimulators of GLP-1 secretion both *in vitro* and *in vivo* and chronic studies should assess their suitability as nutritional therapies for type 2 diabetes.

## Introduction

Glucagon-like peptide-1 (GLP-1) is an incretin hormone primarily produced in the entero-endocrine L-cells of the distal intestine, and physiologically it is secreted in response to nutrient ingestion [1]. GLP-1 is an attractive therapeutic target for type 2 diabetes because of its important actions, including stimulation of insulin biosynthesis and secretion, inhibition of glucagon secretion, inhibition of gastric emptying and acid secretion, reduction of food intake, and expansion of beta-cell mass in the pancreas (see review) [1]. A number of anti-diabetic drugs are now clinically available which act by mimicking or enhancing GLP-1 action, and a new recent focus of research is the identification of compounds which stimulate intestinal secretion of GLP-1. This study investigated the *in vitro* and *in vivo* effects of some natural putative sources of GLP-1-releasing activity.

Extracts from olive leaves are presumed to stimulate GLP-1 secretion due to a naturally occurring compound called oleanoic acid—an activator of TGR5 receptors which are associated with the secretion of GLP-1 [2]. Glutamine is an amino acid which has been demonstrated to stimulate GLP-1 secretion *in vitro* and *in vivo* [3, 4]. Chlorogenic acid, a phenol compound naturally occurring in coffee, has been linked to GLP-1 secretion and reduced risk of type 2 diabetes [5, 6]. Similarly, the milk protein casein has been demonstrated to stimulate GLP-1 secretion in NCI-H716 cells [7]. In this study we tested the acute secretory activity of olive leaf extract (OLE), glutamine (GLN), chlorogenic acid (CGA) alpha-casein (ACAS) and beta-casein (BCAS) in STC-1 cells and went on to assess *in vivo* effects by measurement of responses in mice following an oral glucose tolerance test (OGTT).

## Results and Discussion

**Fig. 1.**
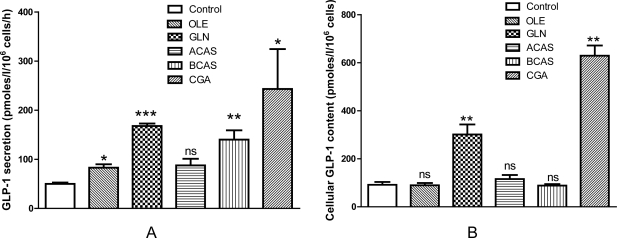
Effects of natural putative GLP-1 secretagogues on GLP-1 secretion and cellular GLP-1 content in STC-1 cells. (A) STC-1 cells were incubated for 3h with OLE, GLN, ACAS, BCAS or CGA before determination of GLP-1 secretion. (B) Cellular GLP-1 content of STC-1 cells following 72 h incubation with OLE, GLN, ACAS, BCAS or CGA, or vehicle control (media). GLP-1 was removed from cells using an acid/ethanol extraction method and CCK concentrations were determined by radioimmunoassay. Results are mean ± SEM (n=6). *P<0.05, **P<0.01 and ***P<0.001 compared with control, ns- not significant. Experiments were repeated on 2 occasions.

**Fig. 2.**
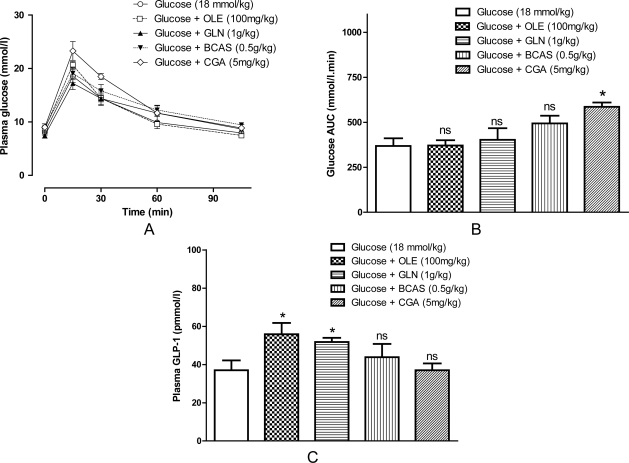
Effects of oral OLE, GLN, BCAS, and CGA on glucose tolerance and GLP-1 concentrations in mice. (A) OLE (100mg/kg), GLN (1g/kg), BCAS (0.5 g/kg), or CGA (5 mg/kg) were orally gavaged with glucose (18 mmol/kg) to mice and glucose responses were monitored at 0, 15, 30, 60 and 105 min. (B) Incremental areas under plasma glucose curves (ΔAUC0-105) were calculated using a computer-generated program employing the trapezoidal rule with baseline subtraction. Area under the curve (AUC) values data were compared using repeated measures one-way Analysis of Variation (ANOVA) followed by the Student-Newman-Keuls post hoc test. (C) Mice were orally gavaged with glucose (18mmol/kg) or glucose in combination with putative GLP-1 secretagogues and after 30 min mice were sacrificed, blood obtained by cardiac puncture and GLP-1 concentrations were determined in plasma. Results are mean ± SEM (n=6); *P<0.05 compared with glucose alone; ns- not significant.

With the exception of ACAS all of the putative secretagogues significantly increased acute GLP-1 secretion compared with vehicle control; and ACAS was not selected for *in vivo* studies. OLE, GLN, BCAS and CGA significantly increased GLP-1 secretion 66%, 236%, 180% and 386%, respectively (Figure 1A; P<0.01–0.001). Interestingly, GLN and CGA also led to significant increases (Figure 1B; 227% and 583%, respectively) in cellular GLP-1 content over the 72h incubation period indicating that these compounds may increase proglucagon gene expression or GLP-1 biosynthesis. Increases in plasma GLP-1 secretion where observed for OLE and GLN (Figure 2C; 48% and 41%, respectively), but surprisingly none of the putative GLP-1 secretagogues reduced AUC values for glucose during OGTTs (Figure 2B).

Type 2 diabetic patients consuming GLN (30g) exhibited significantly higher circulating levels of GLP-1, as well as GIP and insulin, but it is as yet unclear whether GLN improves glycaemic control [4]. Here, we have demonstrated a consistent effect of GLN in stimulating GLP-1 both *in vitro* and *in vivo*. However, during glucose challenge conditions GLP-1 response of GLN did not lead to acute glucose lowering. Casein has previously been reported to stimulate GLP-1 secretion *in vitro* [7, 10]. Casein protein encompasses two protein sub-types: alpha and beta. Our *in vitro* studies indicate that BCAS has greater GLP-1 secretory activity than ACAS. Meal-related challenge of human volunteers with a diet containing 10% or 25% casein demonstrates that compared with other protein sources casein is not as important for eliciting GLP-1 secretion [11]. This study is in good agreement finding no significant alteration in plasma GLP-1 levels following oral administration of BCAS. CGA was the only compound tested which altered OGTT responses, however it rather unexpectedly appeared to increase glycaemic excursions rather than reduce them. A previous study in humans reported that compared with placebo 1g of CGA reduced early glucose responses but not the overall AUC values during the OGTT [12]; furthermore no changes in plasma GLP-1 levels were recorded [13]. We also did not find any evidence that CGA alters GLP-1 concentrations during OGTT.

Other studies have demonstrated that continued consumption of natural putative GLP-1 secretagogues over a prolonged period improves hyperglycaemia associated with diabetes [2, 14]. This study demonstrates that on a cellular level OLE, GLN, BCAS and CGN potently stimulate GLP-1 secretion, but *in vivo* studies only indicate modest rises in plasma GLP-1 concentrations for OLE and GLN. Therefore, the amount of GLP-1 released by these putative GLP-1 secretagogues is perhaps not sufficient to counteract a rapid rise in glucose levels following an oral glucose challenge. In can be concluded that the potential benefits of these GLP-1 secretagogues are not likely to be realised over an acute period, and chronic studies in diabetic animal models should be undertaken to assess their usefulness as nutritional therapies for type 2 diabetes.

## Experimental

OLE was prepared by dichloromethane extraction of powdered olive leaves (Olea europoaea) as previously described [2]. Briefly, dried powdered olive leaves (10g) (Olea europaea) were extracted with dichloromethane (100 mL) using soxhlet apparatus. Solvent was evaporated under reduced pressure and the remaining residue was weighed and stored at −20˚C (for more details see [2]). GLN (>99% purity), CGA (>95% purity), ACAS (>70% purity) and BCAS (>98% purity) were purchased from Sigma (Poole, Dorset, UK). STC-1 cells were obtained from from Dr D Hanahan (University of California, San Francisco, CA) and cultured as previously described [8, 9]. Briefly, cells were cultured in DMEM containing 4.5 g/l D-glucose, (GlutaMAX, GIBCO, Paisley, UK) supplemented with 17.5% foetal bovine serum (FBS), 100U/ml penicillin and 100mg/L streptomycin and incubated in a 5% CO_2_ humidified atmosphere at 37°C. Cells underwent passage upon reaching 80–90% confluence and were used between passage numbers 15–50.

For acute GLP-1 secretion approximately 2×10^6^ cells (n=6) were seeded into 12 well plates and incubated for 18h at 37°C. Media was removed, the cells were washed with HEPES buffer and then underwent 60 min pre-incubation with buffer (20mM HEPES, 10 mM glucose, 140nM NaCl, 4.5mM KCl, 1.2mM CaCl_2_, 1.2mM MgCl_2_). Buffer was aspirated off and cells were incubated for 3h with 400μl of vehicle or OLE (1mg/ml), GLN (40 mM), ACAS (1 mg/ml), BCAS (1 mg/ml), and CGA (40 mM). The responsiveness of cells was verified by conducting test incubations with a mixture of fatty acids as a positive control (data not shown). After the test period 350μl of the incubation solution was removed to a separate tube, placed on ice and centrifuged (900g, 5 min) to remove any cellular debris. The supernatant was collected and stored at −80°C prior to analysis by GLP-1 radioimmunoassay. For cellular GLP-1 content STC-1 cells (1.5 × 10^6^) were seeded into 6 well plates and cultured overnight at 37°C in a humidified atmosphere of 5% CO_2_. Media was removed and fresh media or secretagogue supplemented media was added. Media was removed and replaced with fresh supplemented media every 24h until an incubation period of 72h was reached. Media was then removed, cells were washed and GLP-1 was extracted by the addition of acid/ethanol (1.5% HCl (v/v): 75% ethanol (v/v): 23.5% H_2_O (v/v)) and incubated overnight at 4°C. Acid/ethanol solutions were removed, centrifuged (900g, 5 min) to remove cellular debris, and the ethanol evaporated off using a SpeedVac sample concentrator (Genevac, Ipswich, UK). Samples were reconstituted in buffer prior to radioimmunoassay. GLP-1 was measured using an in-house fully optimised radioimmunoassay which used a GLP-1(7–36)amide standard curve (Molar IC_50_ = 1.6×10^11^; R^2^ = 0.99) and employed anti-rabbit IgG Sac-Cel (IDS, Boldon, UK), and a polyclonal rabbit antibody with no cross-reactivity for glucagon or GIP.

OGTT studies involved 16h-fasted C57BL/6 mice receiving oral gavage of glucose (18 mmol/kg), or glucose in combination with OLE (100mg/kg), GLN (1g/kg), BCAS (0.5 g/kg), or CGA (5 mg/kg). For studies measuring plasma GLP-1, mice were sacrificed in a carbon dioxide atmosphere 30 min post-administration and blood samples taken by cardiac puncture using a heparinised syringe. Blood was centrifuged for 30 seconds at 13000g (IEC Micromax RF) and plasma stored at −80˚C prior to analysis.
